# Study on the Cross-Scale Effects of Microscopic Interactions and Mechanical Properties of Rigid Polyurethane Foam Driven by Negative-Temperature Environments

**DOI:** 10.3390/polym16111517

**Published:** 2024-05-27

**Authors:** Wei Wei, Yusui Bi, Gehua Bi

**Affiliations:** School of Chemistry and Chemical Engineering, Shandong University of Technology, Zibo 255000, China

**Keywords:** rigid polyurethane foam, interaction, molecular simulation, micromechanical properties, cross-scale effects

## Abstract

In order to investigate the cross-scale effects of the interaction between the hard and soft segments of stiff polyurethane foam on the material’s mesoscopic pore structure and macroscopic compression characteristics in various negative-temperature environments, this paper used molecular dynamics to calculate the interaction differences between hard and soft segments in different negative-temperature environments. The effects of various negative-temperature settings on the cell structure of stiff polyurethane foam were investigated using scanning electron microscopy and Image J software. Finally, macro experiments were used to determine the influence of a negative-temperature environment on the characteristics of stiff polyurethane foam (such as compressibility). The molecular simulation calculation results show that in a negative-temperature environment, decreasing temperature gradually increases the interaction between hard segment molecules and soft segment molecules, resulting in an increase in the molecules’ modulus and cohesive energy density. The scanning electron microscope results reveal that a negative-temperature environment gradually increases the pore diameter of stiff polyurethane foam. The compression experiment findings demonstrate that, for the same service duration, the compressive strength in the −20 °C environment is 27.53% higher than that in the 0 °C environment. The study’s findings reveal a microscopic mechanism for the following receiving alterations and toughness enhancement of rigid polyurethane foam throughout service in negative-temperature conditions.

## 1. Introduction

In recent years, rigid polyurethane foam has been widely employed as an insulation material in structures, as well as in building panels and components, due to its exceptional strength and insulating capabilities [1,2,3]. Whether it is used for insulation or building construction, in addition to the functional features of rigid polyurethane foam, its strength must match the criteria of real usage in order to avoid damage and a loss of functionality. As a result, as researchers investigate the functioning of stiff polyurethane foam, they are increasingly shifting their focus to the investigation of its strength qualities [4,5]. Ridha, M., et al. [4] investigated the changes in the interior pores of stiff polyurethane foam when stretched. According to research findings, stretching causes the interior cells of hard polyurethane foam to gradually elongate, compromising its mechanical qualities. Peyrton, J., et al. [6] found that the mechanical characteristics of stiff polyurethane foam are linked to cells. The research results of Wang, Z., et al. [7] show that the effect of water will affect the performance of rigid polyurethane foam. Research conducted by Bhagavathula, K.B., et al. [8] found that the cell structure of rigid polyurethane foam will affect the density and material properties of rigid polyurethane foam.

Furthermore, stiff polyurethane foam is unavoidably impacted by its surroundings, influencing its strength qualities. As a result, researchers investigated the performance of stiff polyurethane foam under different environmental conditions. Wang, J., et al. [9] discovered that temperature affects the mechanical properties of rigid polyurethane foam, but their study only looked at macroscopic properties. It is unclear how the interaction of molecules affects the mechanical properties of rigid polyurethane foam. Hwang, B.K., et al. [10] discovered that temperature impacts the dynamic compression performance of rigid polyurethane foam. Barszczewska-Rybarek, I., et al. [11] investigated the influence of a high-temperature environment on the characteristics of rigid polyurethane foam materials, observed morphological changes in rigid polyurethane foam using scanning electron microscopy, and assessed its flexural and compressive strengths. According to research findings, increased temperature causes unequal cell distribution in rigid polyurethane foam, reducing its flexural and compressive strength.

However, the majority of existing research in this area focuses on the macroscopic changes in the mechanical properties of rigid polyurethane foam in a positive temperature environment, and the structure of the soft and hard segments that comprise rigid polyurethane foam influences the macroscopic mechanical properties of rigid polyurethane foam materials [12,13]. The majority of existing research in this area focuses on how the presence of soft and hard segments affects the characteristics of rigid polyurethane foam [14,15]. There has been little research on how structural changes in the interplay of soft and hard segments impact the performance of rigid polyurethane foam in low-temperature conditions. Furthermore, the microscopic mechanism of the influence of changes in the interaction structure of soft and hard segments on the characteristics of rigid polyurethane foam has yet to be discovered at the molecular level. Molecular dynamics may yield intermolecular interactions and molecular mechanical characteristics by computing molecules’ thermodynamic properties [16,17].

As a result, this paper will employ molecular dynamics to determine the interaction and structural changes between soft and hard segments in stiff polyurethane foam in various low-temperature conditions and obtain the changes in mechanical properties of rigid polyurethane foam in different low-temperature environments. Finally, the microscopic effect mechanism of low temperatures on the cell structure and mechanical characteristics of stiff polyurethane foam was disclosed at the molecular level, and the microscopic mechanism was validated across scales using compression experiments.

## 2. Microsimulations and Macro Experiments

### 2.1. Microsimulation Process and Calculation Indicators

#### 2.1.1. The Process of Model Building

**Figure 1:**
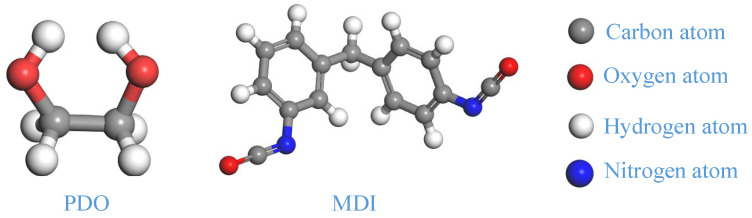
Molecular model of soft and hard segments in rigid polyurethane foam.

The stiff polyurethane foam utilized in this article contains a soft segment of polyoxypropylene dianol (PDO) and a hard segment of methylene diphenyl diisocyanate (MDI). At the same time, the goal of this research is to investigate the influence of interaction structural changes between soft and hard segments on the mechanical characteristics of rigid polyurethane foam. As a result, in this work, to increase the number of molecules in the calculation process, the degree of polymerization of both the soft and hard segments was set to 1. Figure 1 depicts the propylene glycol and MDI molecules created using the Materials Studio program.

**Figure 2:**
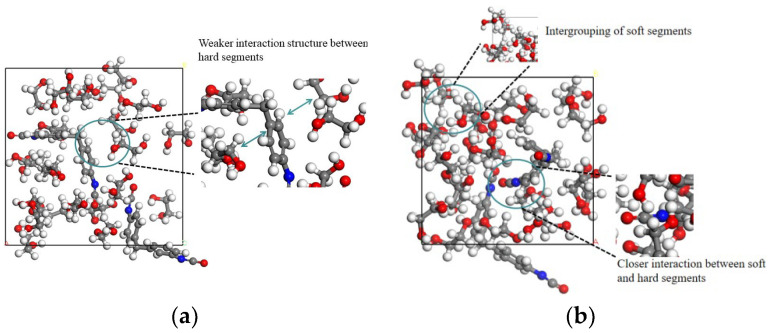
Polyurethane molecules after construction. (**a**) Initial stiff polyurethane molecular model; (**b**) stabilized rigid polyurethane foam model.

Furthermore, we employed the Amorphous Cell module in Materials Studio 8.0 software to create a rigid polyurethane foam molecular model based on the weight ratio of soft and hard segments in the rigid polyurethane foam that we generated. When creating a molecular model to determine the structural changes in the interaction between the soft and hard segments of rigid polyurethane foam in a low-temperature environment, we chose to assemble the monomers of the soft and hard segments in a box rather than polymerizing them into a line with a molecular chain. Table 1 and Figure 2 show the number of PDO and MDI molecules, as well as the initial and stable stiff polyurethane molecules created after operating for 100 ps at room temperature (298.15 K) with the NPT ensemble.

Figure 2 shows that in the first structural model, the stiff polyurethane foam’s soft and hard segments have a weak interaction structure and a high molecular distance between them. After running the model for 100 ps, the distance between molecules steadily decreases, and the soft and hard portions exhibit clear mutual interaction. In addition, soft segment molecules also aggregate with each other, indicating the existence of interactions between soft segments and hard segments.

#### 2.1.2. The Details of Model Running 

To replicate various low-temperature situations, we adjusted the model’s operating temperatures to five levels: 273.15 K, 268.15 K, 263.15 K, 258.15 K, and 253.15 K. The above-mentioned stable model was first subjected to 20,000 ps geometry optimization, then annealed 5 times in the temperature range of 300–500 K, and lastly annealed 5 times in the temperature range of 500–300 K to decrease the energy of the model. Then, using the COMPASS force field and NPT ensemble, each temperature was run for 100 ps to replicate the service process of stiff polyurethane at various temperatures at the same time.

#### 2.1.3. Radial Distribution Function

The radial distribution function (RDF) calculates the likelihood of other atoms that are distributed at a distance r from the center atom. The position of the peak of the radial distribution function determines the kind of contact between the two atoms, while the size of the peak determines the intensity of the connection [18]. The calculation formula of the radial distribution function is shown in Equation (1):

(1) $$g\left(r\right)=\frac{1}{4\rho \pi r^{2}\xi r}\frac{\sum_{t=1}^{T}\sum_{j=1}^{N}\Delta N\left(r\to r+\xi r\right)}{N\times T}$$

where *N* is the total number of molecules; *T* is the total calculation time (ps); *r* is the distance from reference molecule; ΔN is the number of molecules in the system interval; and ρ is the density of the system.

**Figure 3:**
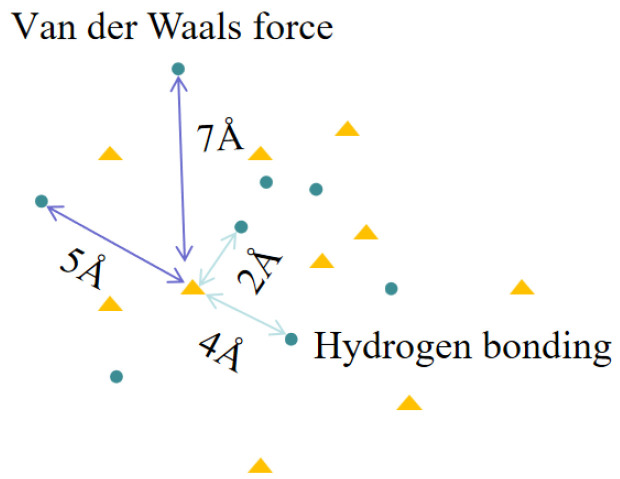
Schematic diagram of hydrogen bonding and van der Waals interactions in radial distribution function.

Figure 3 depicts the link between the radial distribution function and the interaction form of atoms.

#### 2.1.4. Micromechanical Properties

To investigate the micromechanical characteristics of stiff polyurethane foam at various temperatures, this study determines the micromodulus of the system structure after 100 ps of operation in various low-temperature conditions. In molecular dynamics, the static strain technique is commonly used to determine the mechanical characteristics of molecular structures [19]. The elastic stiffness coefficient C_ij_ can be expressed as the following:

(2) $$\left[C_{\mathrm{ij}}=[\begin{array}{llllll} \lambda +2\mu & \;\;\lambda & \;\;0 & \;\;0 & \;\;0 & \;\;0\\ \lambda & \lambda +2\mu & \;\;\lambda & \;\;0 & \;\;0 & \;\;0\\ \lambda & \lambda & \lambda +2\mu & \;\;0 & \;\;0 & \;\;0\\ 0 & 0 & 0 & \mu & 0 & 0\\ 0 & 0 & 0 & 0 & \mu & 0\\ 0 & 0 & 0 & 0 & 0 & \mu \end{array}]\right]$$

where λ and µ are two independent coefficients, and C_ij_ is the stiffness matrix.

The calculation formulas of Young’s modulus (E) and the shear modulus (G) are shown in Equations (3) and (4):

(3) $$E=\mu \frac{3\lambda +2\mu }{\mu +\lambda }$$

G = µ(4)

#### 2.1.5. Cohesive Energy Density

The magnitude of the cohesive energy density can represent the interaction strength, structural stability, and mechanical characteristics of the molecule within itself [20]. The calculation formula is as follows:

CED = coh/V(5)

where CED is the binding energy density (J/cm^3^); coh is the binding energy (J); and V is the simulated volume of the molecule

As a result, we evaluated the cohesive energy density of the molecular structure of stiff polyurethane foam at various temperatures to determine the variations in plastic deformation ability, fracture strength, and fracture toughness.

#### 2.1.6. Calculation of Free Volume

**Figure 4:**
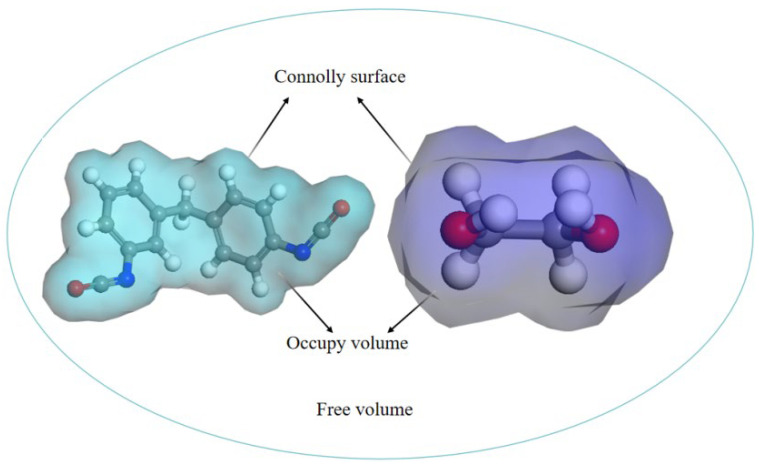
Schematic of the free volume of a molecule.

In molecular dynamics calculations, the free volume of stiff polyurethane molecules can be determined by probe scanning, using MDI and PDO, the basic components of rigid polyurethane foams, as examples (see Figure 4). The barrier between the probe and the atom is specified as the atomic volume surface (Connolly surface). The free volume refers to the volume on the outside of the atomic surface, whereas the occupied volume refers to the volume on the interior of the atomic surface.

This paper will calculate the free volume after running 100 ps in different negative-temperature environments to see if there is shrinkage in the molecular model of rigid polyurethane foam.

### 2.2. Sample Preparation and Experimental Methods

#### 2.2.1. Preparation of Rigid Polyurethane Foam

**Figure 5:**
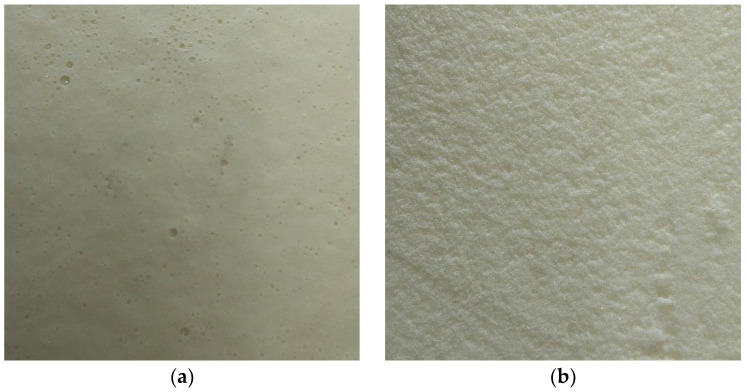
Rigid polyurethane foam specimen. (**a**) Rigid polyurethane foam surface cells; (**b**) rigid polyurethane foam interior.

In this work, propylene glycol (PDO) and diphenylmethane diisocyanate (MDI) were used as raw ingredients to make stiff polyurethane foam. The mass fractions of PDO and MDI were 70% and 30%, respectively, and a catalyst, foaming agent, and stabilizer were added. The raw ingredients were preheated before they were added to the reaction kettle. After the cells formed, the temperature was maintained at a steady point until the firm polyurethane foam was created. The prepared rigid polyurethane foam is shown in Figure 5.

#### 2.2.2. Low-Temperature Treatment of Stiff Polyurethane Foam

Rigid polyurethane foam is commonly employed in low-temperature applications. To assess the effect of low-temperature settings on the mechanical properties of rigid polyurethane foams, we simulated the real use process by subjecting the manufactured specimens to temperatures of 253.15 K (0 °C), 258.15 K (−5 °C), 263.15 K (−10 °C), 268.15 K (−15 °C), and 273.15 K (−20 °C) for 10 days, respectively.

#### 2.2.3. Compression Performance Test

We used an electronic universal material testing machine (CMT-4410, American MTS Systems Company, Eden Prairie, MN, USA) to test the mechanical properties of rigid polyurethane foam at a compression rate of 50% according to GB/T 8813-2020 [21]. The size of the sample was 52 mm × 52 mm × 50 mm, and the compression speed was (5 ± 1) mm/min.

#### 2.2.4. Morphological Testing of Vesicles based on Scanning Electron Microscope (SEM) Experiments

**Figure 6:**
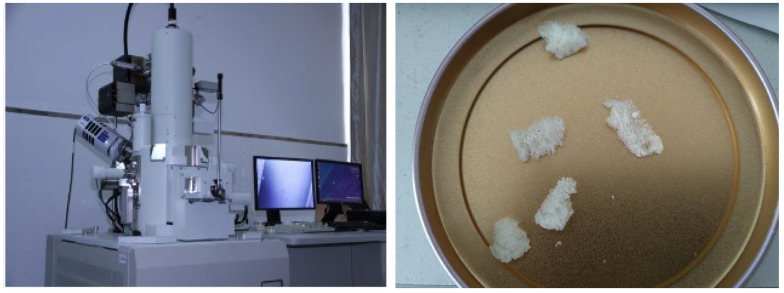
SEM test equipment and specimens.

In this research, the morphology of the vesicles after 10 days of operation at ambient temperatures of 0 °C, −5 °C, −10 °C, −15 °C, and −20 °C was evaluated using a scanning electron microscope. The device utilized was a Japanese JSM-7500F(Nippon Electronics Corporation, Tokyo, Japan) scanning electron microscope with a 25× magnification. The test procedure is shown in Figure 6.

#### 2.2.5. DSC Experiment

**Figure 7:**
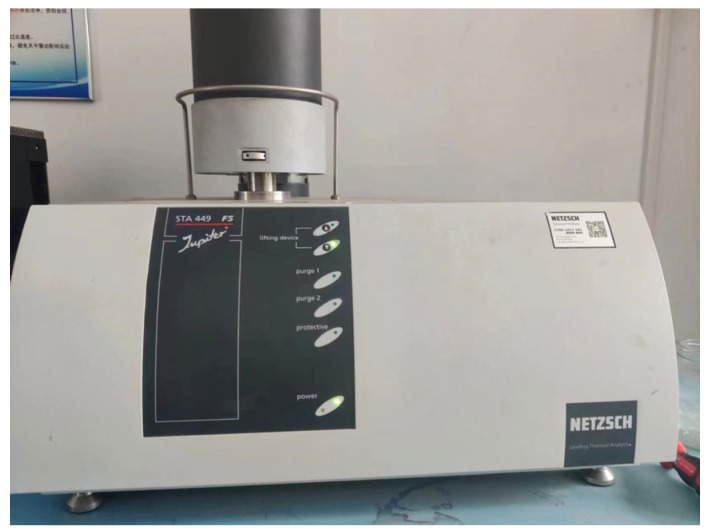
DSC experimental apparatus.

To investigate the effect of the interaction between the structure of adjacent polymer molecular chains in rigid polyurethane foams and the glass transition temperature of rigid polyurethane foams after 10 days of service in a negative-temperature environment, thermogravimetric and differential scanning calorimetry (TG/DSC) experiments were used to test rigid polyurethane foams after service in various negative-temperature environments. The testing procedure was shielded by nitrogen gas, with a temperature increase rate of 20 °C/min and a temperature range of −70 °C to 200 °C. The DSC experimental setup used in this paper was from the German company NETZSCH (Hanau, Germany). The test equipment is shown in Figure 7.

#### 2.2.6. Three-Point Bending Experiments

**Figure 8:**
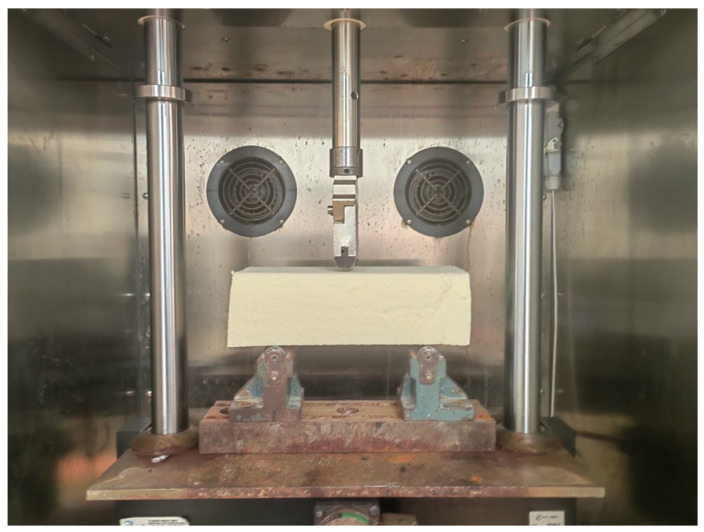
Experimental process of three-point bending.

This paper used a UTM universal testing machine to conduct three-point flexural tests on rigid polyurethane foams after 10 days of service in different negative temperatures to investigate the effect of the interaction between adjacent polymer molecular chains inside the rigid polyurethane foam on the flexural properties of the rigid polyurethane foam after 10 days of service in a negative-temperature environment. The specimen sizes for the experiments were 100 mm × 30 mm × 30 mm, with an impact speed of 2 mm/min. To reduce the possibility of unexpected experimental outcomes, three parallel specimens were created in each negative-temperature environment. The test procedure is shown in Figure 8.

## 3. Results and Analysis

### 3.1. Model Validity Verification

**Figure 9:**
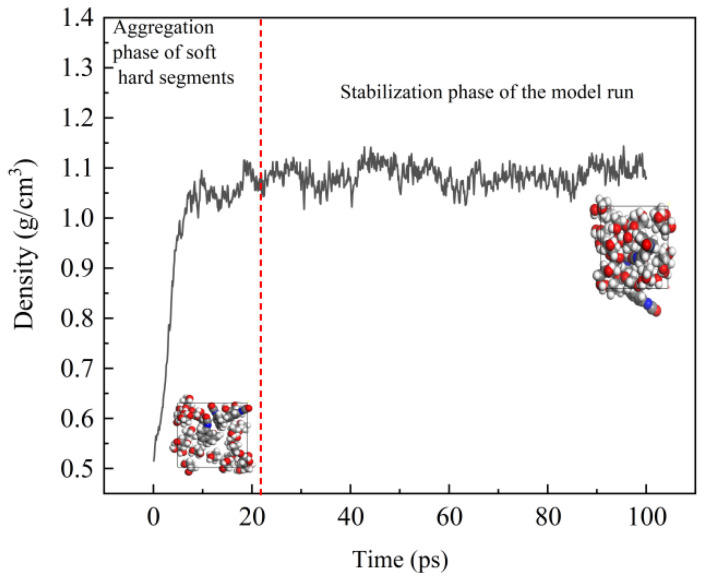
Density and molecular structure changes during model operation.

In molecular dynamics, the model’s validity may be checked using the density once it runs stably. When the simulated density is near to the material’s real density, the created molecular model may be tested as correct [22]. To verify the effectiveness of the constructed rigid polyurethane molecular model, we set the density of the initial rigid polyurethane molecular model to 0.5 g/cm^3^ and ran it under the NPT ensemble for 100 ps to ensure that there was sufficient interaction between the hard and soft segments, and then the density after the model stabilized was compared to the density of the actual rigid polyurethane. Figure 9 depicts how the stiff polyurethane molecular model’s density and molecular structure change during the process of running simulations.

Figure 9 shows that the distance between the soft and hard segments of the rigid polyurethane molecules is quite good in the early stage, and the soft and hard segment molecules are significantly scattered. After running for 100 ps, the contact between the soft and hard segments brings the soft and hard segment molecules closer together, finally producing dense polyurethane molecules. The stabilized density is approximately 1.1 g/cm^3^, while the actual density of polyurethane ranges from 0.9 g/cm^3^ to 1.2 g/cm^3^ [23]. As a result, it is possible to demonstrate that the built model may be utilized to represent hard polyurethane molecules.

### 3.2. Analysis of Model Running Results

**Figure 10:**
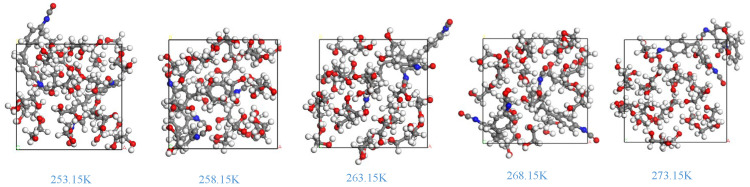
Running results of rigid polyurethane molecular model at different temperatures.

Figure 10 depicts the structural state of the stiff polyurethane molecular model at five temperatures after the NPT ensemble runs for 100 ps.

The operational condition of the model at various temperatures in Figure 10 shows that as the temperature rises, the space between molecules progressively increases, and the structural state of the stiff polyurethane molecules gradually changes from “compact” to “dispersed”. According to the report, this is because rising temperatures force molecules to travel faster, causing hard and soft segment molecules to gradually separate. It is also possible that a rise in temperature may reduce the connection between soft segments, hard segments, and soft–hard segments, resulting in changes in the characteristics of rigid polyurethane molecules.

### 3.3. Analysis of Free-Volume Calculation Results

**Figure 11:**
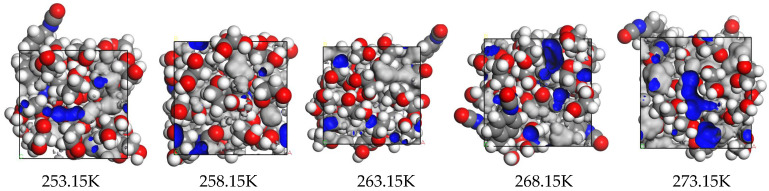
Free volume of rigid polyurethane molecules at different temperatures.

To confirm the above conjecture regarding the changes in the molecular structure of stiff polyurethane at different temperatures, this study determined the free volume at different temperatures, as shown in Figure 11.

This article determined the free-volume percentage of the stiff polyurethane molecular structure model using Formula (6):

FFV = V_f_/(V_f_ + V_0_) (6)

where FFV is the free-volume fraction (%); V_f_ is the free volume; and V_0_ is the volume occupied by molecules.

**Figure 12:**
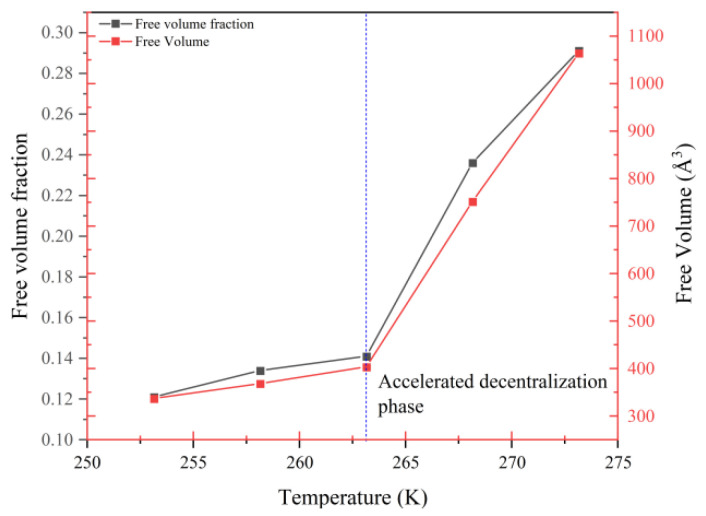
Free volume and free-volume fraction of rigid polyurethane foam molecules at different temperatures.

Finally, Figure 12 depicts the free volume and free-volume percentage of stiff polyurethane foam at various temperatures.

Figure 12 shows that when the temperature rises, the free volume and free-volume percentage in the stiff polyurethane molecular structure model increase. This confirms the change in the molecular model structure shown in Figure 10, which suggests that as the temperature drops, the soft and hard segments in the rigid polyurethane molecules aggregate with each other, and it also shows that when the temperature is lower, the degree of molecular aggregation is greater, and the hard segments and pores between soft segment molecules become smaller.

### 3.4. Analysis of the Effect of Temperature on Soft–Hard Segment Interactions

**Figure 13:**
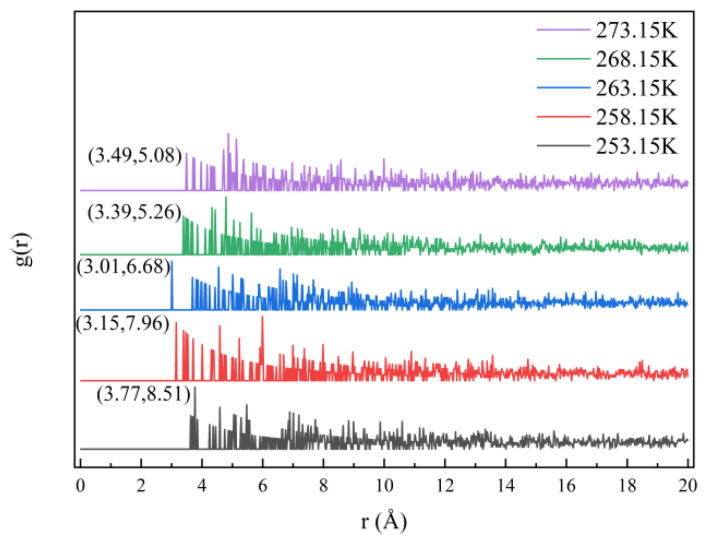
Interaction intensity of soft and hard segments at various temperatures.

Calculating the radial distribution function yields the interaction shape and strength between molecules [24]. We computed the stiff polyurethane molecular model’s radial distribution function in various low-temperature conditions because the stiff polyurethane molecule has a connection between the PDO hydroxyl group and the MDI isocyanate group [25]. During the computation stage, the oxygen and nitrogen atoms were picked in MDI. The calculation results are shown in Figure 13.

Figure 13 shows that the radial distribution functions of hard and soft segments in rigid polyurethane foam at five temperatures exhibit maxima in the 3-4Å range. And starting from 0 °C (273.15 K), as the temperature decreases, the peak value gradually increases. Relevant investigations have revealed that for nitrogen and oxygen atoms, the distinctive peak of the radial distribution function emerges within this region, suggesting that a covalent connection exists between oxygen and nitrogen atoms at this moment [26]. Lowering the temperature enhances the interaction between hard and soft segments, allowing the hard and soft segment molecules in polyurethane to combine with one another.

### 3.5. Analysis of Cohesive Energy Density Calculation Results

**Figure 14:**
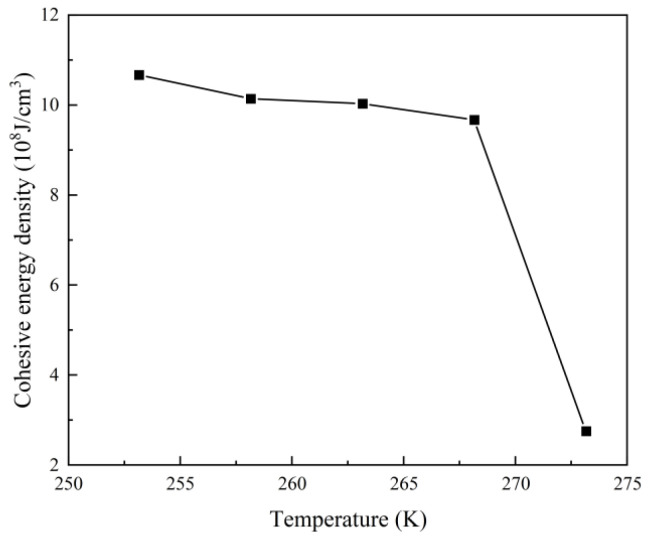
Cohesive energy density of rigid polyurethane foam molecules at different temperatures.

The cohesive energy density might indicate a material’s fracture resistance. The higher the cohesive energy density, the better the material’s fracture resistance [27]. As a result, in order to assess the effect of low-temperature environments on the fracture resistance of rigid polyurethane foam, we estimated the cohesive energy density of rigid polyurethane molecular models in various low-temperature conditions. The calculation results are shown in Figure 14.

The findings in Figure 14 show that stiff polyurethane molecules have the highest cohesive energy density at 253.15 K (−20 °C) and the lowest cohesive energy density at 273.15 K (0 °C). There is a 74.13% difference between the two. The change rate of cohesive energy is minimal between 253.15 K and 268.15 K, but between 268.15 K and 273.15 K, the cohesive energy density drops dramatically by 71.45%. It may be concluded that reducing the temperature enhances the cohesive energy density of stiff polyurethane foam. This article speculates that this is connected to the level of intermolecular interaction seen in Figure 13. When the temperature is low, the soft and hard segments of stiff polyurethane molecules have greater interactions, boosting cohesive energy.

This article speculates that the sudden drop in cohesive energy density in the temperature range of 268.15 K to 273.15 K is due to a change in the glass transition temperature of rigid polyurethane molecules after they are exposed to various low-temperature environments for an extended period of time. Relevant research has shown that after placing the polymer material at a lower temperature for a period of time, the internal molecular arrangement structure becomes more orderly and compact, and increasing the temperature accelerates the thermal motion of the molecules, resulting in a difference in glass transition temperature [28]. Therefore, when the temperature is less than 268.15 K, the hard polyurethane molecules act in a glassy state, and the molecular structure exhibits high hardness. When the temperature exceeds 268.15 K, the rigid polyurethane molecules react in a stretchy manner. Because the elasticity is high at this point, the cohesive energy density is the lowest. Furthermore, the glass transition temperature of genuine stiff polyurethane foam ranges between 253.15 K (−20 °C) and 313.15 K (40 °C). This article speculates that the disparity between the glass transition temperature predicted from molecular simulation results and the actual glass transition temperature is due to the molecular model that was created. Rigid polyurethane foam is composed of soft and hard segments that polymerize into polyurethane monomers. These monomers aggregate by intermolecular van der Waals forces, hydrogen bonds, and Π-Π stacking interactions [29]. The molecular model that we created illustrates the interaction of soft and hard segments in separate nearby polyurethane molecular chains, with just the soft and hard segment monomers being generated. This creates a glass transition temperature that is distinct yet similar to the glass transition temperature of the true stiff polyurethane foams.

### 3.6. Analysis of the Impact of a Negative-Temperature Environment on the Micromechanical Characteristics of Stiff Polyurethane Foam

**Figure 15:**
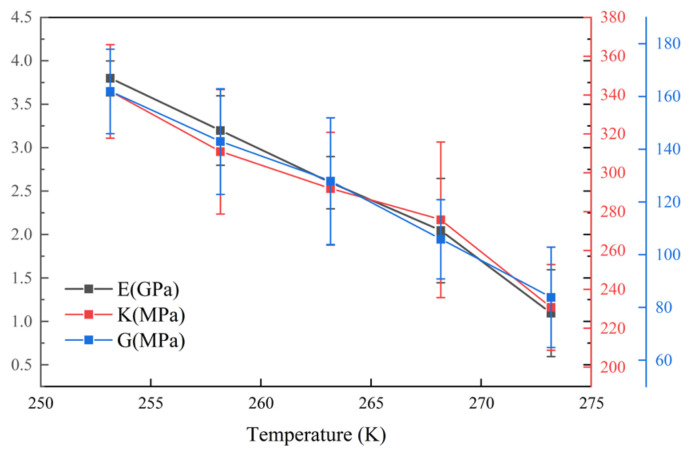
Calculation results of the molecular modulus of rigid polyurethane at different temperatures.

In materials science, the bulk modulus (K), Young’s modulus (E), and the shear modulus (G) can all indicate a material’s mechanical characteristics. The bigger the bulk modulus, the stronger the volume stability of the material; the larger the Young modulus, the better the elastic characteristics of the material; and the larger the shear modulus, the stronger the shear resistance of the material [30]. This study evaluated the modulus of stiff polyurethane molecules in various low-temperature conditions. Figure 15 displays the results of the calculations.

Figure 15 shows that at lower temperatures (such as 253.15 K), stiff polyurethane molecules have a greater Young modulus, bulk modulus, and shear modulus. As the temperature rises, the mechanical characteristics of stiff polyurethane molecules gradually deteriorate. When compared to 253.15 K, the Young modulus, bulk modulus, and shear modulus at 273.15 K fell by 71.05%, 32.46%, and 48.15%, respectively. As a result, it is possible to conclude that storage in a low-temperature environment can dramatically raise the Young modulus of stiff polyurethane materials and, to a lesser degree, the bulk modulus, although only slightly. This is consistent with the previously indicated shifting trends in the interaction strength and cohesive energy density of stiff polyurethane molecules, and this reflects changes in material attributes. In this instance, when served or stored at a lower temperature, the interaction between the soft and hard segments in rigid polyurethane gradually increases, as does the material’s hardness and compression resistance.

### 3.7. Analysis of Changes in the Morphology of Microscopic Vesicles in Rigid Polyurethanes

**Figure 16:**
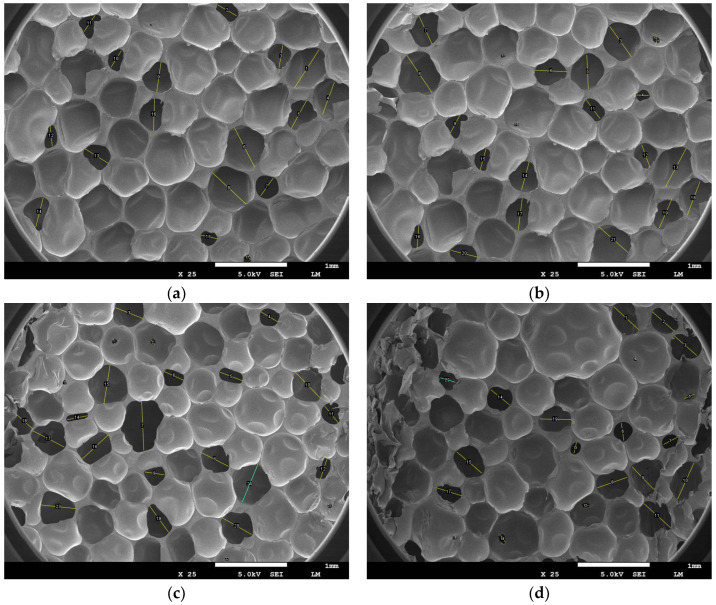

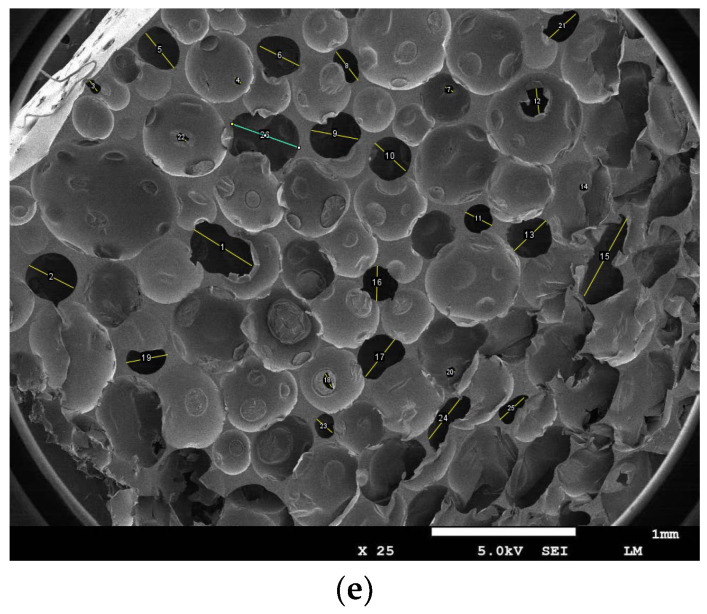
Pore morphology of stiff polyurethane foam under various negative-temperature conditions: (**a**) 273.15 K, (**b**) 268.15 K, (**c**) 263.15 K, (**d**) 258.15 K, and (**e**) 253.15 K.

The size of the vesicles can indicate the shrinkage changes in rigid polyurethane foams when used in low-temperature situations. The bubble forms of the rigid polyurethane foam after 10 days of service at different temperatures are shown in Figure 16.

Figure 16 shows that after ten days of service at 273.15 K, the rigid polyurethane foam cells had grown larger. As the temperature drops, the cells gradually contract. After serving at 268.15 K for the same period, micropores emerged, and after serving at 263.15 K, 258.15 K, and 253.15 for the same time, the cells continued to shrink and additional micropores appeared.

This article speculates that this effect is caused by the fact that under negative-temperature settings, as the temperature falls, the distance between neighboring polymer chain structures within the stiff polyurethane foam is reduced, resulting in increased interaction between the two. The improved contact results in the macroscopic shrinkage of stiff polyurethane foam.

In order to express this phenomenon quantitatively, we used Image J software to calculate the number of pores and the average pore diameter of rigid polyurethane foam in different negative-temperature environments, as shown in Table 2:

Table 2 demonstrates that when the temperature decreases, the average pore diameter of rigid polyurethane foam decreases. The average pores at the temperature of 253.15 K decreased by 33.5% compared with that at 273.15 K. In addition, when the temperature ranges from 273.15 K to 263.15 K, the number of pores in the scanned area shows an increasing trend. This article speculates that this is due to the temperature-induced shrinking of stiff polyurethane foam, as additional pore structures can be seen under the same magnification. However, when the temperature is 258.15 K, the number of pores decreases. This article speculates that this is due to a reduction in temperature and the closing of certain small pores during stiff polyurethane foam shrinking. When the temperature lowers to 253.15 K, the number of pores in the region rises while maintaining the same service time and magnification factor. This demonstrates that, even when tiny holes are closed, the hard polyurethane foam continues to shrink at lower temperatures, resulting in additional pores becoming visible at the same scanning magnification.

To summarize, in a negative-temperature environment, when the temperature decreases, stiff polyurethane foam shrinks progressively, resulting in reduced pore diameters and the closing of microscopic holes. This will result in a tighter structure of stiff polyurethane foam, affecting its macroscopic characteristics. It also confirms the veracity of the simulation results in Figure 8 and Figure 9.

### 3.8. Analysis of the Impact of Temperature on the Macroscopic Compression Characteristics of Stiff Polyurethane Foam

**Figure 17:**
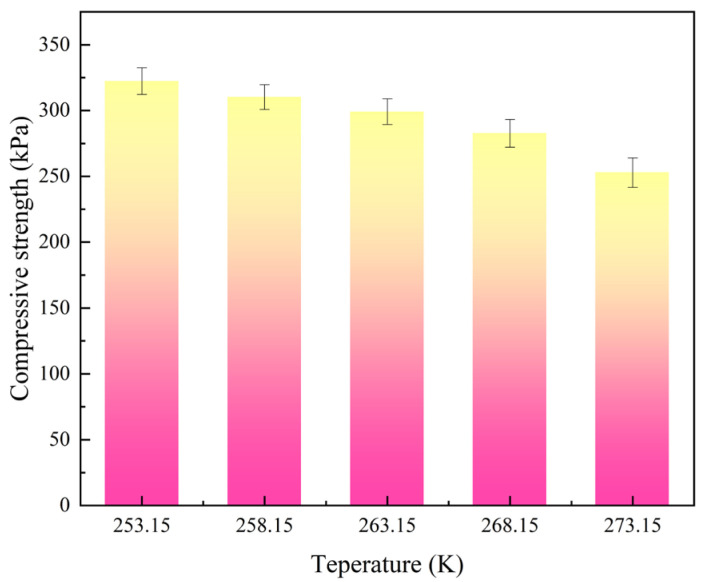
Compressive strength of rigid polyurethane foam at different temperatures.

In order to explore the impact on the compressive properties of rigid polyurethane foam after serving in different low-temperature environments for the same time, we tested the compressive strength of rigid polyurethane foam after serving in different low-temperature environments for 10 days. The test results are shown in Figure 17.

**Figure 18:**
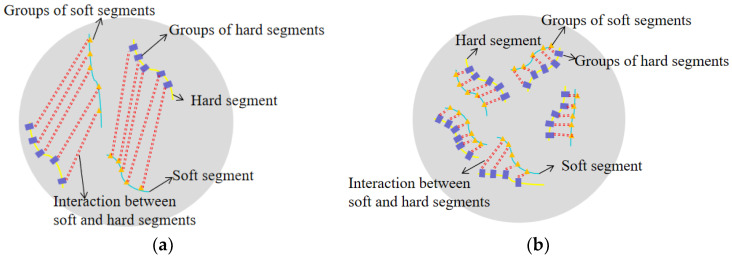
Schematic depiction showing the change in force intensity between stiff polyurethane molecules in various low-temperature settings. (**a**) Intermolecular interactions at 0 °C; (**b**) intermolecular interactions at −20 °C.

The findings in Figure 17 show that when temperature falls, the compressive strength of stiff polyurethane foam steadily rises. At 268.15 K, the compressive strength change rate is the highest at 11.8%. After 10 days in a 253.15 K environment, compressive strength rises by 27.53% compared to 10 days in a 273.15 K environment. This article speculates that this is related to the “crystalline-like” behavior of the polymer molecules of rigid polyurethane foam at low temperatures. In a low-temperature environment, polymer molecules will aggregate and shrink with each other, causing the polymer molecules to behave like crystals in the material. The results of the molecular dynamics model also show that the increase in temperature causes the soft segment and hard segment molecules in the rigid polyurethane structural model to be dispersed. Therefore, the molecules in the rigid polyurethane material aggregate with each other, shortening the distance between molecules and increasing the interaction force between molecules (a schematic diagram is shown in Figure 18). When bearing external loads, it has a stronger reaction force to resist external loads, thus improving the compressive strength of rigid polyurethane foam. At the same time, this is consistent with the research results of Seymour, R.W., et al. [31]. 

Figure 18 shows that the shrinkage of stiff polyurethane foam increases as the temperature decreases. This results in an increase in the intermolecular forces of the polyurethane material and a weakening of molecular mobility, showing stronger compressive resistance when subjected to the same external load.

**Figure 19:**
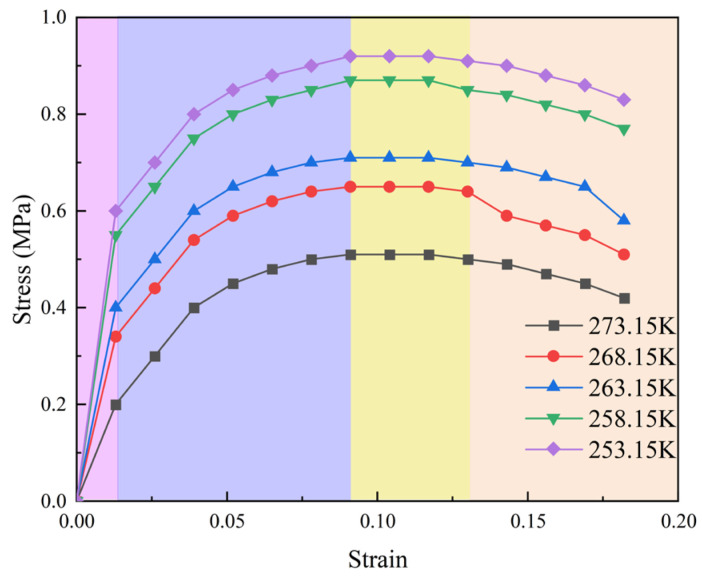
Stress–strain curve.

It can be seen from the data in Figure 19 that the stress–strain curves of rigid polyurethane foam are different after being stored in different low-temperature environments for a period of time. The elastic phase of the stress–strain curve can reflect the elastic modulus of the material [32]. Therefore, it can be judged that the elastic modulus of rigid polyurethane foam after 10 days of storage in a 253.15 K environment is the largest, while the elastic modulus of rigid polyurethane foam after 10 days of storage in a 273.15 K environment is the smallest. This is consistent with the results of the molecular simulation mentioned above in this article; that is, the lower the temperature, the stronger the interaction between hard segment molecules and soft segment molecules in rigid polyurethane foam, and the tighter the overall rigid polyurethane molecular structure. In addition, it can be seen from the stress–strain curve that the compressive strength of rigid polyurethane foam in a 253.15 K environment is significantly higher than that in a 273.15 K environment, which is consistent with the rule shown in Figure 14.

### 3.9. Analysis of Bending Performance Test Results

**Figure 20:**
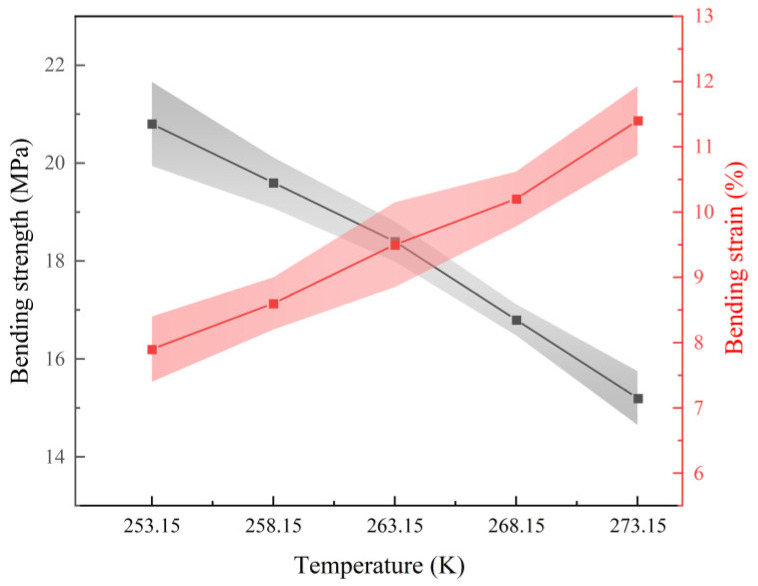
Flexural qualities of stiff polyurethane foam after 10 days of use in various negative-temperature conditions.

Figure 20 depicts the test results for the flexural strength and flexural strain of rigid polyurethane foam after 10 days of service in various negative-temperature settings.

Figure 20 shows that while the service duration is the same, the flexural strength of stiff polyurethane foam declines, but the flexural strain increases with temperature. The bending strength in the 253.15 K environment was 26.92% higher than in the 273.15 K environment, while the bending strain fell by 30.7%. The bending strength in the 253.15 K environment was 26.92% higher than in the 273.15 K environment. It is clear that in a negative-temperature environment, when the temperature drops, the plastic deformation capacity of stiff polyurethane foam diminishes, brittleness rises, and bending characteristics improve. This article hypothesizes that this is due to the drop in temperature, which leads to an increase in the contact between surrounding polymer chain structures inside the stiff polyurethane foam (schematically depicted in Figure 17). The increased contact causes the shrinkage of rigid polyurethane material (as illustrated in Figure 16), and the rigid polyurethane foam becomes more compact, resulting in better flexural characteristics. 

### 3.10. Analysis of DSC Test Results

**Figure 21:**
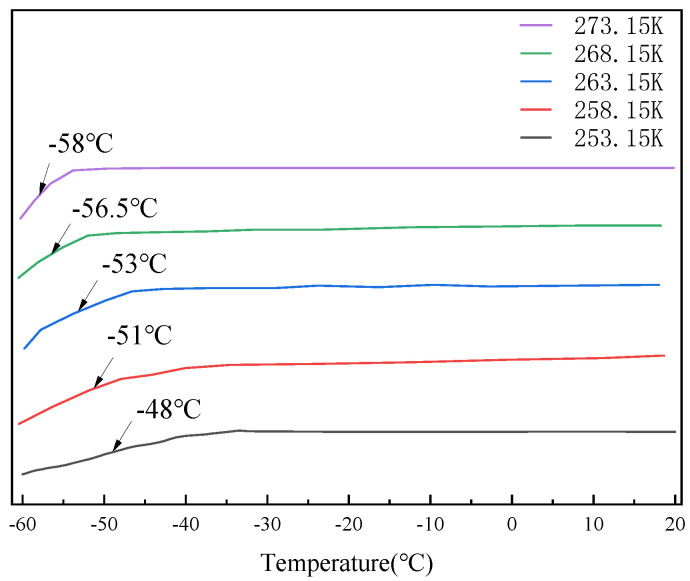
Glass transition temperature of rigid polyurethane foam in different negative-temperature service environments.

After testing, we discovered that the glass transition process of the stiff polyurethane foam that we manufactured occurs within the temperature range of −60 °C to −40 °C. To further demonstrate the variation in the glass transition temperature of rigid polyurethane foam after serving in different negative-temperature settings for the same duration, we used the DSC test data in the temperature range of −60 °C to 20 °C. Furthermore, to distinguish between the negative ambient temperature and the glass transition temperature acquired from DSC experimental testing, the ambient temperature ranged from 253.15 K to 273.15 K, while the glass transition temperature ranged from −58 °C to −48 °C. The test results are shown in Figure 21.

Figure 21 shows that the lower the ambient temperature of the service, the higher the glass transition temperature of the stiff polyurethane foam when the service period remains constant. In this article, it was theorized that this happened because, as the temperature steadily decreases, the contact between the chains of surrounding polyurethane molecules inside the stiff polyurethane gradually increases, causing the material to contract. Rigid polyurethane molecules tend to become increasingly ordered, which increases the stability and deformation resistance of rigid polyurethane foams. Therefore, higher temperatures are needed to weaken the intermolecular interactions and realize the glassy state transition. This results in the occurrence of the stiff polyurethane foams’ glass transition temperature steadily increasing as the temperature decreases when used in a negative-temperature environment. 

### 3.11. Analysis of the Cross-Scale Effects of Rigid Polyurethane Foam Micro-Interactions Rigid Polyurethane Foam

**Figure 22:**
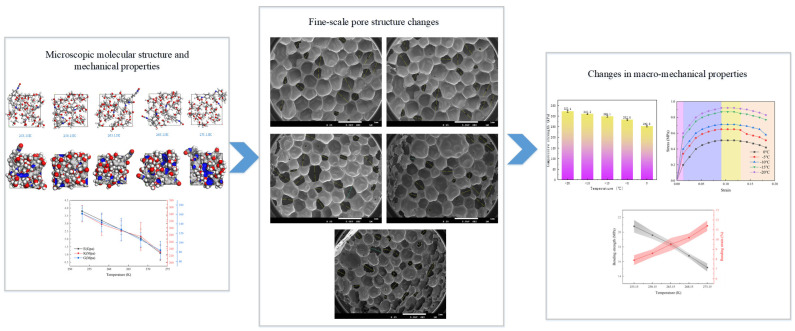
Cross-scale effects of shifting interactions between soft and hard segments on the structure and mechanical characteristics of rigid polyurethanes.

In summary, the interaction between the soft and hard segments in rigid polyurethane foams affects their service performance in different environments. In negative-temperature conditions, the connection between the molecules of the soft and hard segments is more tightly organized, resulting in a steady increase in hardness. The relationship between the effect of specific micro-interactions on the macroscopic compression properties is shown in Figure 22.

As shown in Figure 22, the interaction between the soft and hard segments of the rigid polyurethane molecule gradually increases as the temperature lowers in a negative-temperature environment, resulting in the contraction of the molecular structure. The best molecular mechanical characteristics of stiff polyurethane foam can be observed at temperatures ranging from 253.15 K to 273.15 K. Furthermore, the contraction of the molecular structure resulted in changes to the fine pore structure, as well as to the macroscopic compressive properties, revealing the mechanism of the influence of the microscopic molecular structure change on the fine structure and macroscopic mechanical properties of the rigid polyurethane foam.

## 4. Conclusions

This paper uses molecular dynamics to calculate the interaction differences between soft segments and hard segments in rigid polyurethane foams in different negative-temperature environments. The effect of negative temperatures on the interaction between soft segment molecules and hard segment molecules was proposed, and the cross-scale impact of differences in interaction on the cell structure and compression properties of rigid polyurethane foam was analyzed. The conclusions that we reached are as follows:

(1) In a negative-temperature environment, as the temperature decreases, the hard segment molecules and soft segment molecules in rigid polyurethane foam aggregate more obviously, which will make adjacent rigid polyurethane molecular chains more likely to entangle and cross-link with each other.
(2) The calculation results of the radial distribution function show that in a negative-temperature environment, as the temperature decreases, the intensity of interaction between hard segments and soft segments gradually increases, which will cause adjacent rigid polyurethane molecular chains to become more entangled with each other for closeness.
(3) The calculation results of cohesive energy density and micromechanical properties show that in a negative-temperature environment, as the temperature decreases, the strength and modulus of rigid polyurethane molecules will gradually increase.
(4) The scanning electron microscopy test results reveal that the diameter of the blister holes of the rigid polyurethane foam gradually increases with a decreasing temperature in the negative-temperature environment due to the contraction of the rigid polyurethane molecular chains that aggregate with each other, confirming the accuracy of the molecular simulation results.
(5) The compression performance test results show that in a negative-temperature environment, as the temperature decreases, the compression performance of rigid polyurethane gradually increases, which is consistent with the results of microscopic simulation.

To sum up, during service in a negative-temperature environment, the interaction between the soft segments and hard segments of rigid polyurethane foam will gradually increase, which will lead to a gradual increase in the strength of the material. The aim of this article is to reveal the cross-scale effects of intermolecular interactions and structural changes in rigid polyurethane foam on the microscopic pores and macroscopic properties of rigid polyurethane foam. However, it is worth paying attention to the resistance to the fracture of materials in low-temperature environments. This will be the focus of subsequent research on this topic.

## Figures and Tables

**Table 1:** Molecular formulas and number of molecules of PTMG and MDI.

Molecule Name	Molecular Formula	Number of Molecules	Simulated Quality Score	Actual Quality Score
PDO	HO-(CH_2_)_2_-OH	21	72.25%	70%
MDI	C_15_H_10_N_2_O_2_	2	27.74%	30%

**Table 2:** Pore number and pore size in different negative-temperature environments.

Temperature/K	273.15	268.15	263.15	258.15	253.15
Number of pores	17	21	22	19	25
Average pore diameter/mm	0.391	0.368	0.331	0.296	0.260

## Data Availability

Data are contained within the article.
